# General anesthesia in a patient with asymptomatic second-degree two-to-one atrioventricular block

**DOI:** 10.1186/s40981-017-0099-0

**Published:** 2017-05-10

**Authors:** Marie Shigematsu-Locatelli, Takashi Kawano, Atsushi Nishigaki, Daiki Yamanaka, Bun Aoyama, Hiroki Tateiwa, Noriko Kitaoka, Masataka Yokoyama

**Affiliations:** Department of Anesthesiology and Intensive Care Medicine, Kochi Medical School, Kohasu, Oko-cho, Nankoku, Kochi 783-8505 Japan

**Keywords:** Atrioventricular block, Cardiac pacing, Anesthesia

## Abstract

**Background:**

The major perioperative concern in patients with second-degree atrioventricular (AV) block is the progression to complete AV block. Therefore, the prophylactic implantation of a temporary pacemaker prior to surgery is recommended, especially in symptomatic patients. However, as no quantitative preoperative risk assessment from progression to complete AV block is available, there is currently no established indication for preoperative prophylactic pacemaker implantation. Here, we present a case of progression from asymptomatic second-degree two-to-one (2:1) AV block to complete AV block following the induction of general anesthesia.

**Case presentation:**

A 69-year-old female with degenerative spinal stenosis was scheduled for transforaminal lumbar interbody fusion surgery under general anesthesia. She had no cardiac symptoms, but routine preoperative resting 12-lead electrocardiogram revealed second-degree 2:1 AV block. After discussion with the surgeon and referring cardiologist, we scheduled the surgery without implantation of a temporary pacemaker before surgery for the following reasons: (1) asymptomatic, (2) no evidence of underlying cardiac disease, and (3) a narrow QRS complex. On the day of surgery, general anesthesia was induced with 150 mg of intravenous thiamylal and 25 μg of fentanyl, followed by intravenous administration of 50 mg of rocuronium to facilitate endotracheal intubation. Sevoflurane (1.0–2.0%) was used to maintain anesthesia. A few minutes after induction, the 2:1 AV block progressively converted to complete AV block, and the surgery was postponed. During emergence from anesthesia, the third-degree AV block recovered to 2:1 AV block, similar with the preoperative pattern. The patient was monitored in the intensive care unit for 2 days and then transferred to the normal orthopedic ward uneventfully. One month later, the surgery was rescheduled with preoperative implantation of a temporary pacemaker. A slow mask induction using sevoflurane with oxygen was started. Upon loss of consciousness during the inhalation of initial sevoflurane, complete AV block developed and temporary pacing was immediately initiated. Subsequent anesthesia and surgery were uneventful. The patient made an uncomplicated recovery from surgery with stable hemodynamics. The temporary pacemaker was not required after surgery, and the pacemaker catheter was removed 1 day after surgery.

**Conclusions:**

The present case indicates that a prophylactic pacemaker should be implanted preoperatively in patients who have 2:1 AV block even without symptoms.

## Background

Second-degree atrioventricular (AV) block is characterized by an intermittent interruption of impulse transmission from the atria to the ventricles [1]. Based on the electrocardiogram pattern, second-degree AV block is classified into type I (Mobitz I or Wenckebach; progressive prolongation of the PR interval until non-conducted P wave occurs), type II (Mobitz II; constant PR interval until non-conducted P wave occurs), or two-to-one (2:1) AV block [1, 2]. Type I AV block commonly occurs within the AV node, and confers a benign prognosis, especially in case of no underlying heart disease. On the other hand, type II AV block may be located infranodal and has the potential to progress to complete AV block. Clinical practice guidelines consistently recommend that permanent cardiac pacing is indicated in patients with type II AV block [3, 4]. However, in 2:1 AV block, there is one PR interval before the blocked P wave making impossible to distinguish between type I and type II block [2, 5]. Therefore, the indication for permanent pacing remains sometimes controversial in patients with 2:1 AV block, unless it causes symptoms or occurs at the infranodal level.

High grade AV block is a major predictor of perioperative cardiac complications, especially significant bradyarrhythmias, in non-cardiac surgery [6]. In the perioperative period, multiple factors may contribute to the progression of incomplete AV block to complete AV block [7]. Although temporary cardiac pacing may be urgently required as definitive therapy if complete AV block develops, the indication for prophylactic temporary cardiac pacing before surgery remains unclear. In addition, there is no published report regarding anesthetic management of patients with 2:1 AV block. Here, we described a case of a transient complete AV block during induction of general anesthesia in a patient with asymptomatic fixed 2:1 AV block.

## Case presentation

**Fig. 1:**
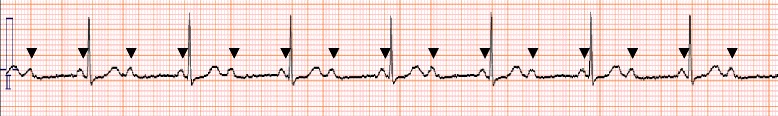
Preoperative electrocardiogram. The tracing of lead II shows second-degree atrioventricular block with 2:1 AV conduction, ventricular rate of 46 beats per minute. Every other P wave (*marked*) was regularly conducted. Note the short PR interval during conducted complexes and the narrow QRS complexes

A 69-year-old female (weight 41 kg, height 142 cm) with degenerative spinal stenosis was scheduled for transforaminal lumbar interbody fusion surgery under general anesthesia in prone position. She was not taking any medication and had no known previous cardiovascular disease, as well as no family history of sudden cardiac death. In addition, the patient had no cardiovascular symptoms, including syncope, tachycardia, or chest pain, under at least 4 METs of daily activity. However, routine preoperative resting 12-lead electrocardiogram (ECG) revealed a second-degree 2:1 AV block with a narrow QRS complex (0.102 s), heart rate (HR) of 46 bpm, normal axis, and with no ischemic change (Fig. 1). The patient was sent to the cardiology department in our hospital for detailed cardiac examinations. Holter’s ECG recording for 24 h indicated the constant fixed 2:1 AV block. Echocardiography demonstrated a normal left ventricular systolic function with ejection of 80.2% with no segmental anomaly. At that time, exercise stress testing and cardiac artery computed tomography were not conducted due to the symptom of lumbar spinal canal stenosis and patient’s denial, respectively. After discussion with the consulting cardiologists, we concluded that she was at a low risk of progression to high grade AV block as she was asymptomatic, had a stable heart rhythm, had no established underlying cardiac disease, and a narrow QRS interval. Therefore, we decided that a temporary (or permanent) pacemaker would not be implanted before surgery in this case. In addition to the general preoperative explanation, the attending anesthesiologist informed the patient about the potential risks associated with her AV block, as well as our recommendations including relevant alternatives, obtaining the consent for the management of anesthesia.

**Fig. 2:**
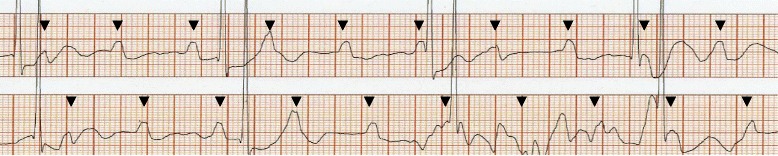
Intraoperative monitor electrocardiogram following induction of general anesthesia. The traces show complete atrioventricular block with junctional escape rhythm of approximately 30–40 beats per minute. The P waves (*marked*) were completely dissociated from the QRS complexes. Artifacts most likely due to poor electrode contact with the skin caused the noisy baseline in this tracing

On the day of surgery, no premedication was administered before surgery. Standard monitoring systems, including ECG, pulse-oximetry, and non-invasive blood pressure, as well as a radial arterial line, were placed in the operating room. An external transcutaneous pacemaker was ready to use in case it was needed. Just before induction of anesthesia, the patient’s ECG pattern remained similar to preoperative examinations, i.e., second-degree 2:1 AV block, at a rate of 46 bpm, and blood pressure was 121/74 mmHg, SpO_2_ was 97% at room air. After administration of 100% oxygen, general anesthesia was induced with 150 mg of intravenous thiamylal and 25 μg of fentanyl followed by intravenous administration of 50 mg of rocuronium to facilitate endotracheal intubation. Sevoflurane (1.0–2.0%) was used to maintain anesthesia. A few minutes after induction, the HR gradually decreased to <40 bpm. Atropine was then given intravenously as a bolus of 0.5 mg, but showed ineffective. Conversely, the 2:1 AV block progressively converted to complete AV block (Fig. 2). During the third-degree AV block, the hemodynamic collapse did not occur, i.e., the patient’s hemodynamic status was relatively stable with a HR of 38–46 bpm and BP of up to 80/50 mmHg. Arterial blood gas analysis revealed no pathological data. Therefore, the pacing or pharmacological interventions were not considered at that time. After discussion with the surgeons and cardiologists, surgery was postponed in order to implant prophylactic transvenous pacemaker leads and to obtain an additional patient’s informed consent. Subsequently, sevoflurane inhalation was interrupted and 200 mg of sugammadex was given for reversal of neuromuscular block. During emergence from anesthesia, the third-degree AV block recovered to 2:1 AV block, similar to the preoperative pattern. The patient emerged smoothly and was extubated in the operating room. The vital signs remained stable with HR in the range of 48–61 bpm and BP in the range of systolic 100 to 138 and diastolic 68 to 80 mmHg. She was monitored in the intensive care unit for 2 days and then transferred to the normal orthopedic ward uneventfully.

The patient’s surgery was rescheduled for 1 month later. This time, implantation of a preoperative transvenous temporary pacemaker was performed for the emergent treatment of bradyarrhythmias, with the patient agreement. On arrival to the operating room, the patient’s ECG showed 2:1 AV block, same as the previous time, at a rate of 52 bpm, and blood pressure was 119/67 mmHg. A slow mask induction using sevoflurane with oxygen was started. Upon loss of consciousness during the inhalation of the initial 2% sevoflurane, HR decreased to <40 bpm just as it did during the first anesthesia. Temporary pacing was immediately initiated with 60 bpm in VVI mode (output 5 mA, sensitivity 1 mV), after that it worked well. Subsequent anesthesia and surgery were uneventful. The emergence from anesthesia was smooth, and she was extubated without difficulty in the operating room. The patient made an uncomplicated recovery from surgery with stable hemodynamics. Since the temporary pacemaker was no longer required, the catheter was removed 1 day after surgery. The patient was discharged 14 days postoperatively and has been in periodical follow-up at the department of cardiology. Approximately 6 months after surgery, the patient experienced bradycardia-related symptoms such as dizziness and weakness and underwent permanent pacemaker implantation. Her symptoms completely disappeared after the implantation.

## Discussion

The major perioperative concern in patients with cardiac conduction abnormalities is the potential risk for progression to complete AV block during surgery [6]. The development of a sudden complete AV block requires urgent pacing intervention. However, intraoperative insertion of transvenous pacing lead may be difficult due to the surgical positioning of the patient. Accordingly, in general, preoperative implantation of a prophylactic temporary pacemaker should be considered as mandatory in high-risk patients like those with symptomatic type II second-degree AV block or sick sinus syndrome. In other cases of conduction disturbances, however, the optimal indication of prophylactic pacemaker insertion prior to surgery has long been contentious and remains unestablished [6, 8]. In the present case, the patient presented a second-degree fixed 2:1 AV block, but was asymptomatic without evidence of underlying cardiac disease. After discussion with the surgeon and referring cardiologists, we scheduled the surgery without implantation of a permanent pacemaker, as well as prophylactic temporary leads, before surgery. Unfortunately, the patient developed a complete AV block following induction of general anesthesia, and as a consequence, the surgery was postponed.

The prognosis of patients with second-degree AV block depends on the anatomic levels of blockage [1, 2, 9]. A conduction abnormality within the AV node (nodal block) is commonly benign, whereas a block distal to the AV node (infranodal block) represents a high risk of progression to complete heart block. Hence, the current clinical guidelines recommend a class IIa indication for permanent pacing in patients with the second-degree AV block at infranodal levels, regardless of symptoms [5]. Electrocardiographically, nodal and infranodal block are typically associated with type I and type II block, respectively. As 2:1 AV block can be either nodal or infranodal, determination of the anatomic site in 2:1 AV block is an important clinical decision when making the therapeutic strategy. Generally, it may be distinguished by the width of the QRS complex; a narrow QRS complex suggests nodal block, while a broad QRS complex indicates an infranodal block [1, 2, 9].

The QRS complex of our patient was narrow, and we expected the nodal (type I) second-degree 2:1 AV block to have a low risk of inadvertent complete AV block. However, it is not always possible to determine the site of AV block without electrophysiological evaluation, e.g., an atypical case of narrow QRS complex type infranodal AV block [9]. Furthermore, in our case, intravenous injection with atropine did not increase, but paradoxically decreased in the conducted ventricular rate. This response is a well-known phenomenon in patients with infranodal AV block [9]. In retrospect, these clinical findings imply that the location of the AV block in our patient may have been at the level of infranodal.

In order to make a definitive diagnosis of the anatomical site of the AV blockade, bundle recordings during cardiac electrophysiological study are needed [10, 11]. However, as these recordings are invasive, it is usually performed in selected patients with symptomatic arrhythmias of unknown origin. Nevertheless, the Japanese guidelines for non-pharmacotherapy of cardiac arrhythmias has further assigned class IIa recommendation for electrophysiological study in patients with asymptomatic AV block who need treatment with drugs that may exacerbate conduction disturbance [10]. On the other hand, currently, there is no consensus whether preoperative patients meet this criterion. During the perioperative period, the AV nodal conduction system may be affected by many factors, such as anesthetics, alternations of blood gases or electrolytes, or surgical manipulation [7, 8]. Therefore, in preoperative patients with suspicious infranodal AV conduction abnormalities, like our presented case, cardiac electrophysiological studies should be conducted. Otherwise, the prophylactic transvenous pacemaker leads should be temporarily implanted before anesthesia. In order to validate this point of view, further clinical evidence is needed.

Other than the transvenous technique, transcutaneous and transesophageal cardiac pacing are also available during an emergency as a temporary pacemaker [12]. Transcutaneous pacing is a non-invasive external pacing technique, but it does not provide for AV synchronized pacing and it is unsuitable during surgery due to skeletal muscle stimulation [13]. Transesophageal pacing is generally recommended only for atrial pacing, and it cannot be applied to patients in prone position [14]. Accordingly, transvenous pacing is considered a more reliable way to provide atrial, ventricular, or AV pacing in surgical patients. However, transvenous pacing is technically more difficult to perform and has the potential for more complications related to the venous access and lead placement [15]. Therefore, unnecessary routine prophylactic insertion of the transvenous pacing lead should be avoided.

In our case, complete AV block rapidly developed following induction of anesthesia with thiamylal and sevoflurane in the first and second surgery, respectively. Previous preclinical studies suggested that clinically relevant concentrations of anesthetics generally have no or moderate negative dromotropic effects on AV conduction [16, 17]. However, clinical data regarding the effects of anesthetic agents on cardiac electrophysiology, especially in patients with infranodal abnormalities, are lacking. Although it is unknown whether the complete AV block observed in our case was caused by a direct electrophysiological effect or a nonspecific autonomic effect, we highlight the importance of inserting a prophylactic transvenous pacemaker in patients with infranodal AV block.

## Conclusions

Here, we reported a case of progression to complete AV block following induction of general anesthesia in a patient with asymptomatic fixed 2:1 AV block. The risk of progression from 2:1 AV block to complete AV block depends on whether the site of the block is nodal or infranodal. However, it cannot always be confirmed without performing cardiac electrophysiological study. By the present case, we emphasize that a prophylactic pacemaker should be implanted preoperatively in patients who have 2:1 AV block even without symptoms.

## Abbreviations

AV: Atrioventricular
ECG: Electrocardiogram
HR: Heart rate
